# Transgender women’s perspectives on mental health care related to vaginoplasty for gender affirmation

**DOI:** 10.1186/s12905-023-02681-w

**Published:** 2024-01-03

**Authors:** Emily E. Marra, Hilary Mabel, Sharon Feldman, Mary Beth Mercer, Murat Altinay, Cecile A. Ferrando

**Affiliations:** 1 Case Western Reserve University School of Medicine, Cleveland, OH USA; 2 Clinical Ethics, Wellstar Health System, Marietta, GA USA; 3 University of Melbourne, Melbourne, Australia; 4 Center for Bioethics, Women’s Health Institute, Cleveland Clinic, Cleveland, OH USA; 5 Department of Psychiatry and Psychology, Center for Behavioral Health, Center for LGBT Care, Cleveland Clinic, Cleveland, OH USA; 6 Center for Urogynecology and Pelvic Reconstructive Surgery, Obstetrics & Gynecology Institute, Cleveland, OH USA

**Keywords:** Transgender, Mental health care, Qualitative methods, Vaginoplasty

## Abstract

**Purpose:**

This study aimed to describe patient experiences and attitudes about the role of the mental health professional as it relates to pursuing gender affirmation surgery.

**Methods:**

This was a mixed-models study with semi-structured interviews. Participants who presented for gender affirming vaginoplasty and had completed pre-surgical requirements but had not yet had the procedure were invited to participate in the study. Semi-structured phone interviews were conducted from November 2019 and December 2020 until saturation of themes was achieved at a sample size of 14. Interviews were then transcribed verbatim and coded by theme. Qualitative analysis was performed using a grounded theory approach.

**Results:**

Almost half of the patients did not identify any barriers to obtaining mental health care, but a majority brought up concerns for less advantaged peers, with less access to resources. Some patients also felt that there was benefit to be obtained from the mental health care required before going through with surgery, while others felt the requirements were discriminatory. Finally, a large proportion of our participants reported concerns with the role of mental health care and the requirements set forth by the World Professional Association for Transgender Health (WPATH), and patients gave suggestions for future improvements including decreasing barriers to care while rethinking how guidelines impact patients.

**Conclusion:**

There are many competing goals to balance when it comes to the guidelines for gender affirmation surgery, and patients had differing and complex relationships with mental health care and the pre-surgical process.

## Introduction

Transgender patients are a growing population with unique healthcare needs, which for many transgender women includes access to gender affirming surgeries like vaginoplasty [1, 2]. In 2015, the US Transgender Survey reported that 10% of transgender women had undergone a prior vaginoplasty or labiaplasty while an additional 45% reported that they desired to undergo the surgery in the future [3].

Transgender women seeking vaginoplasty undergo a unique pre-surgical process that is outlined by the World Professional Association for Transgender Health Standards of Care (WPATH SOC), an international document that provides a clinical framework for the care of transgender patients around the world. In this framework patients are asked to submit two referral letters from two separate “qualified mental health professionals” [1] prior to being approved for genital gender affirmation surgery (two letter requirement) [4].

There is currently a robust debate on the role and impact of the two letter requirement, whether it creates barriers to care or provides support and ensures needed mental health care for a population with higher rates of mental health needs than cisgender peers [5–7].

Therapy and mental health assessment prior to surgery is theorized to lead to better outcomes for patients by providing mental health treatment for patients that they might not receive otherwise [8]. Some argue, however, that the WPATH SOC letter requirement unduly restricts patient autonomy, may reflect professional and cultural discomfort with treating gender dysphoria [9] and unnecessarily pathologizes the experience of being transgender [10]. There have also been arguments that difficulties obtaining letters can create barriers to or delay treatment, and that the requirement is unnecessarily invasive [11, 12].

What has been missing from the conversation is more information coming directly from transgender patients about their experiences and perceptions of the WPATH SOC. Therefore, the purpose of this study is to examine the experience of transgender women who have successfully navigated the pre-requisites needed for vaginoplasty surgery.

## Methods

### Patient selection and recruitment

This study was performed with Cleveland Clinic Institutional Review Board approval. Patients were included if they presented to the Cleveland Clinic for vaginoplasty, if they were at least 18 years of age, English speaking, and had met both the WPATH SOC for vaginoplasty surgery as well as institutional guidelines, and had not yet had the procedure. All patients who were scheduling vaginoplasty surgery during the study data collection dates were referred to the research team, and those patients who met the above criteria were contacted.

Candidates were sent a cover letter explaining the study and were given the option to opt out from further contact. Those who did not opt out were contacted via telephone to discuss study participation. Participants were recruited sequentially, and contact was attempted a total of five times and then ceased. No voicemails were left out of concern for participant privacy. A total of twenty-five candidates were contacted: five did not answer phone calls, two declined to participate, three failed to return consent forms, and one candidate’s consent form arrived too late to schedule an interview pre-operatively. 14 total participants were recruited due to early thematic saturation. Interviews were conducted between November 2019 and December 2020. Participants received a $25 gift card for their participation.

### Data collection

A semi-structured interview guide was developed with a panel of experts with relevant clinical and methodologic expertise after a review of the literature. The interview guide contained a series of open-ended questions addressing how patients engaged with mental health care, what their experiences with mental health care and obtaining referral letters were like, their perceptions of the pre-surgical process, and how the pre-surgical process could be improved as it relates to mental health care. Verbal and written informed consent was obtained and documented prior to conducting interviews, which were audio recorded, transcribed verbatim, and verified by a second researcher. The transcripts were de-identified and stored on a password-protected computer on a secure server only accessible to the research team performing the interviews and participating in their transcription. The participant’s medical and surgical team was blinded to participant involvement in the study, and these individuals did not have direct access to study interviews or transcripts.

Participant demographics and social history were obtained at the end of the semi-structured interview. Data were stored and managed electronically using REDCap (Research Electronic Data Capture), a secure, web-based application designed to support data input for research studies.

### Data analysis

Qualitative analysis was performed using a directed content analytical approach. This was conducted as an iterative and progressive process of data immersion, coding, memoing, and theme identification. The interview guide, along with a sample of five transcripts, was used to create an initial coding tree based on concept domains set out in the interview guide and codes identified in the initial sample of transcripts. The coding tree was continuously modified as additional codes were identified in the data. Each transcript was coded independently by two members of the research team (EM, SF).

As additional codes were identified and added to the coding tree, previously coded transcripts were reviewed for the existence of these additional codes. The data analysts met regularly to review coded data for consistency and to identify themes. An experienced qualitative researcher (MBM) assisted with the coding and analysis process. Any coding discrepancies between EM and SF were resolved after discussion with a third team member (HM or MBM). The research team met regularly to review data coding and memos, to identify themes, and incorporate clinical perspectives throughout the analytic process. Throughout the recruitment process, there was continuous evaluation for thematic saturation, or the point at which collection of further data by recruitment of additional research participants did not yield new insights. This was reached earlier than anticipated after 14 participants, and thus recruitment was ended.

For the quantitative portion of the analysis, descriptive statistics were computed; no comparisons were done. All data were presented as n (%). Jmp v15.0 was used for this analysis. For the qualitative portion of the analysis, coded themes were tabulated and also presented as n (%).

## Results

Fourteen participants were enrolled in the study and underwent the semi-structured interview. Participant characteristics are shown in Table 1. The mean age of participants was 34 (± 14) years and the majority identified as white (92.9%) and were highly educated, with 71.4% holding a bachelor’s or graduate degree. While 85.7% of the participants reported a diagnosis of depression, the majority of participants reported good family or friend supports for their transition (71.4%).

**Table 1:** Participant characteristics (*N* = 14)

Characteristic	Value
Age	34 (14)
Race/Ethnicity	
White/Caucasian	13 (92.9)
Black	0 (0)
Asian	0 (0)
American Indian or Alaska Native	0 (0)
Pacific Islander	0 (0)
Hispanic or Latino	0 (0)
Other	1 (7.1)
Education	
Grade School	0 (0)
High School	0 (0)
Some College, Technical School or Associate’s Degree	4 (28.6)
Bachelor’s Degree	7 (50)
Graduate Degree	3 (21.4)
Sexual Orientation	
Asexual	0 (0)
Heterosexual	0 (0)
Homosexual	4 (28.6)
Bisexual	4 (28.6)
Pansexual	1 (7.1)
Unsure	1 (7.1)
Other	4 (28.6)
Partner Status	
Single	6 (42.9)
Partnered	7 (50)
Other	1 (7.1)
Other Gender Affirming Surgery	5 (35.7)
Chest Surgery	2 (14.3)
Facial Surgery	2 (14.3)
Orchiectomy	4 (28.6)
Other	0 (0)
Tobacco Use	
Current	0 (0)
Previous	4 (28.6)
Non-Prescription Drug Use	2 (14.3)
History of Drug Use	6 (46.2)
Mental Health	
Depression	12 (85.7)
Bipolar Disorder	2 (14.3)
Post-traumatic Stress Disorder	2 (14.3)
History of Homelessness	1 (7.1)
History of Abuse	
Physical	4 (28.6)
Emotional	7 (50)
Sexual	4 (28.6)
Family Support for Transition	
Supportive	10 (71.4)
Unsupportive	4 (28.6)

All data presented as mean (standard deviation) and n (%)

### Patient experiences

#### Prior engagement with mental health care

The majority of participants (57.1%) were already engaged with a mental health care provider prior to seeking letters of referral for surgery; just over one third (36%) of participants engaged with mental health care for the first time in order to obtain their letters for surgery. Of the five participants who first sought out mental health care to get documentation for surgery, two continued to see a provider after obtaining that documentation, and one participant planned to re-engage in mental health care after surgery.

#### Identifying mental health providers

Participants identified three primary methods for finding mental health providers: online search (64.3%), referral from a healthcare professional (50%), or a recommendation from a friend or loved one (28.6%). While most participants only needed to see two providers to obtain their two letters for surgery (64.3%), four (21.4%) participants had to see between three and five providers to get their two letters. One individual whose original letters expired reported having to see up to eight mental health providers to complete the two letter requirement.

#### Barriers to obtaining mental health care

Over half of participants identified a barrier to obtaining care (57%). Table 2 outlines the challenges participants encountered when attempting to obtain mental healthcare, which included lack of specialty providers (36%), long wait time to appointments (36%), and difficulty identifying specialty providers (26%). Participants also reported encountering providers who were unwilling to see transgender patients (14%). One participant described the experience of engaging with mental health care as follows: “It was difficult at first, trying to get a mental health provider to be honest, and then I had to wait to see them. I kind of got really discouraged about that... There just aren’t enough people.” (Participant 8). It was also a challenge to find the kinds of specialty providers participants were seeking, as one explained: “It was not easy to find someone that really had a trans specialty” (Participant 6) Another participant expressed frustration with navigating the process, stating: “it feels like anyone who can slow you down does. Everyone’s not working against you, but on accident, …they’re all screwing it up” (Participant 10).

**Table 2:** Patient identified challenges to obtaining mental health care

Theme	Participant Response
Lack of specialty providers	5 (36)
Long wait time to appointments	5 (36)
Difficulty identifying specialty providers	4 (26)
Providers unwilling to see transgender patients	2 (14)
Expense	2 (14)
Insurance	1 (7)
None for this participant	6 (43)

Data are n (%)

#### Barriers to obtaining referral letters for surgery

Participants identified a number of barriers to obtaining letters of referral for surgery (Table 3). Participants explained the time intensive nature of the process of obtaining documentation for surgery (29%), with long waiting periods (36%). One participant stated: “Getting there is a very difficult process... It’s a years-long process that takes a lot of driving, a lot of money, a lot of time, time off work.” (Participant 11) Participants described providers who were inexperienced caring for transgender patients (21%) or resistant to writing letters for surgery (21%). Participants also described experiencing confusion about what credentials the letter writers needed to have (14%), issues with the expense of the process (21%) and issues with insurance coverage (14%).

**Table 3:** Patient identified challenges to obtaining letters of referral for surgery

Theme	Participant Response
Long waiting period for appointments	5 (36)
Time intensive	4 (29)
Providers inexperienced caring for trans patients	3 (21)
Resistance from providers	3 (21)
Expense	3 (21)
Insurance issues	2 (14)
Confusion about letter writer requirements	2 (14)
Expiration of letters	1 (7)
None for this participant	3 (21)

Data are n (%)

Table 4 displays ease of finding two mental health professionals to write letters, as rated using a Likert scale. Eight (57%) participants described the process as easy or somewhat easy, two (14%) as neutral, and four (29%) described it as difficult or somewhat difficult. Notably, eight (57%) participants felt that the two letter requirement created barriers for other patients that they did not experience themselves. As one participant stated: “I think the guidelines are really more written for someone like me who lives in a large city and has the means to acquire all this stuff…For someone who lives an hour southeast of me, it’s not only annoying, it’s potentially, ‘I don’t know if I can get the letters to have the surgery.’” (Participant 1).

**Table 4:** Ease of finding two mental health providers to write letters for surgery

Response	Participant Count
Easy	5 (36)
Somewhat easy	3 (21)
Neutral	2 (14)
Somewhat difficult	2 (14)
Difficult	2 (14)

Data are n (%)

Furthermore, barriers to meeting the letter requirement resulted in treatment delays for some participants. Five (35.7%) participants reported that the timing of their surgery was delayed due to the length of time it took to obtain their letters.

### Benefits of mental health care

Most participants (93%) were able to identify at least one way that the mental health care they received was helpful to them. The most common benefits of pre-surgical mental healthcare identified by participants were related to the therapeutic relationship participants had with providers and included emotional support for transitioning (85.7%), having someone with whom to talk (64.2%), affirmation (50%), and a neutral person with whom to speak (42.9%). Participants also noted tangible benefits including medication to treat psychiatric illness (28.6%), dealing with past trauma (28.6%), coping strategies (28.6%), and confidence for coming out or transitioning (28.6%). Other themes that emerged were having someone with whom to talk who is educated about trans issues (14.3%), normalization of feelings (14.3%), gaining perspective (14.3%), connection to resources (14.3%), alleviation of feelings of fault (7.1%), and connection to mental healthcare if future needs arise (7.1%).

### Unhelpful experiences with mental health care

Forty-three percent of study participants identified ways in which mental health care was not helpful to them, including discomfort with providers (21%), providers being inexperienced in caring for transgender patients (14%), that it was repetitive and without benefit to see multiple providers (14%), and that this care was a “waste of time” (14%).

### Participant perceptions

#### The WPATH SOC

Nearly all participants (92.9%) reported being familiar with the WPATH SOC, with only one participant (7.1%) reporting not being familiar with them. Participants were asked what they presumed to be the purpose of the guidelines. Of the 12 participants who answered this question, 75% identified preventing regret as a goal of the guidelines, ensuring emotional readiness for surgery (42%), ensuring patients had all relevant clinical information (33%), and establishing a standard of care (17%).

Participants’ perceptions of the WPATH SOC were varied. A total of six participants (42.9%) expressed their perspective that these guidelines amount to gatekeeping, a word that participants themselves used, along with other terms such as “jumping through hoops,” “roadblock,” and “unnecessary hurdle.” Four participants (28.6%) expressed a perspective that gatekeeping is done by the medical profession, while three participants (21.4%) expressed a perspective that gatekeeping is done by insurance companies (with one person identifying gatekeeping by both).

One participant described a favorable view of the guidelines: “And I feel like having these like hurdles,... you’re lessening the chances that you involve a person who is going to experience some level of regret in that decision. And that’s for the betterment of the patient number one, but the community number two.” (Participant 7). Three others (21%) expressed skepticism that a person would regret gender affirmation surgery. One participant stated, “I kept trying to explain to everybody, who’s gonna want to do this just because and then change their mind after it’s done?... Who would do that?” (Participant 8).

Participants expressed their frustration with other downsides of the perceived gatekeeping nature of the guidelines: “Society requires infinitely too much of transgender [and] questioning individuals, and the requirements placed on them are vastly more than anyone else seeking surgery... [T]here’s just way too many [expletive] requirements on trans individuals, and even people who are questioning.” (Participant 13). Of note, several participants (28.6%) felt that the WPATH SOC do not appropriately account for non-binary identities. “I feel like there’s some holdover language and holdover concepts from the 90 s, when that was definitely a more, when even being trans was thought about in a very binary scope.” (Participant 7).

#### The two letter requirement

Participants’ perceptions of the two letter requirement are presented in Table 5. More than half expressed that the requirement creates barriers for patients that participants did not experience personally (57%), though 21% experienced barriers themselves. Some participants also expressed that the mental health providers qualified to write the letters were not necessarily the experts in this context (14%) and that they were the true experts on themselves and what is best for them (21%). This theme is illustrated in the following quote: “But seriously if you do need a letter, I’m the expert on me. It just hurts that my opinion isn’t good enough, but these other people who have no idea what they’re doing, if you can convince them that you’re transgender... they are in fact qualified to write it.” (Participant 10). Some participants did express that the two letter requirement provided some benefit (27%). One participant (7%) perceived that the two letter requirement prevents regret.

**Table 5:** Patient perception of two letter requirement

Theme	Participant Response
Creates barriers for others (not experienced by participant)	8 (57)
Gatekeeping	7 (50)
Specific degree requirements for letter writers unnecessary	4 (27)
Provides Benefit	4 (27)
“I am the expert on me”	3 (21)
Created barriers for patient	3 (21)
Transportation	1 (7)
Limited mental health provider availability	3 (21)
Expense	2 (14)
Misapprehension of expertise in this context	2 (14)
12 month expiry of letters unreasonable	2 (14)
Prevents regret	1 (7)

Data are n (%)

Of the 12 participants who gave their perspective on what an ideal number of letters would be, two (17%) participants felt there should not be any letter requirements, five (42%) participants felt only one letter should be required, and three (25%) participants supported the two letter requirement. Several participants also expressed the desire for the process to be changed completely.

#### Ideal process

Participants identified a number of modifications to the presurgical process for vaginoplasty as relates to mental health care, summarized in Table 6. These included more accessible information about providers and their services (29%), more providers who identify as transgender or LGBT themselves (21%), better coordination between different services (21%), decreased costs (21%), requiring an established relationship with only one provider (14%), increased provider availability (14%), and increased flexibility regarding requirements of the WPATH SOC based on individual circumstances (7%). Three (21%) participants preferred having no requirements at all, while three (21%) would want to maintain the current process. One participant advocated for no letters stating “I think there should be a different system entirely. So I guess no letters. Because I think that requiring letters, like I described, is pretty homophobic to begin with.” (Participant 13).

**Table 6:** Patient recommendations for changes to mental healthcare requirements in the pre-surgical process

Theme	Participant Response
More accessible information about available providers and services	4 (29)
Better coordination of different services	3 (21)
Decreased cost	3 (21)
More transgender and LGBTQ providers in healthcare	3 (21)
Increased provider availability	2 (14)
Minimum number of visits or established relationship with just one provider	2 (14)
Flexibility in guidelines for individual circumstances	1 (7)
**No requirements relating to mental health care**	**3 (21)**
**No improvements needed to current process**	**3 (21)**

Data are n (%)

### Additional insights

Participants shared many other insights over the course of the interviews, some of which are illustrated by Table 7. Many participants commented on navigating their transition, one stating “[I]t’s probably one of the hardest things that a human being can navigate.” (Participant 6). Another participant added: “There’s all these hidden rules” (Participant 10).

**Table 7:** Additional illustrative quotes

Theme	Quote
Reasons for engaging in mental health care initially	*“My story has not been seeking out a mental health care professional to get me approved with the letter. That is not at all … My story is, I need help for myself, for mental health, gender dysphoria, dysmorphia, these whole things that they’re here for” (Participant 11)* *“Literally the only reason is the bureaucracy of the state requires me to see someone to get two different letters before I can have surgery. So I did that.” (Participant 1)* *“I just was going there not so much for my own benefit, but to get the letters for surgery.” (Participant 3)*
Diverse and opposing views of the pre-surgical process	*“I couldn’t really expect it to be any better because I thought it was actually pretty easy. The hardest part was to get up enough nerve to seek help” (Participant 2)* *“So it doesn’t sound that bad but hopefully today with all the stuff I said, you see it’s not like a series of soft blows. It is like a traumatizing violent, you’re talking about one of the most vulnerable of your population, who’s probably fired if they ever did get a job. To say that it isn’t violent. It’s all so sad.” (Participant 10)*
Opinion on ideal number of letters	“*Two letters? [Sigh.] I don’t understand that. I guess you could argue that it’s this need for making sure you don’t just have a therapist out there who’s just writing anybody who wants a letter a letter. But I personally feel like two letters feels like a step we don’t need. And a PhD holder also feels like a step, that’s just very, setting up a roadblock. Right, getting, being able to see a therapist is potentially, in at least our healthcare landscape, a luxury for folks.” (Participant 7)* *“[Y]ou should not need any letters for this... They need to change it. It is so degrading... You needed no letters to get $100,000 of student loan debt. Zero letters. You need zero letters to sign up for the Army. Zero. So there’s no reason to have a letter.” (Participant 10)*
Attitudes towards second letter writer	*“The other one felt like much more of a formality. I had to track down someone who met those requirements, and then see them several times, until they [emphasis] were comfortable enough to say that they could write the letter. So that was much more of a formality, of like, rehashing of a lot of the things that I had organically talked about with my primary therapist over the last couple of years. And then I had to bring up [emphasis] this new therapist up to speed and then we could talk about things that mattered for this letter.” (Participant 7)* *“I enjoyed that actually, it’s comforting. It’s definitely necessary because you personally need that outside opinion on, ‘how do you feel about this?’” (Participant 12)*
Mental health providers inexperienced with caring for transgender patients	*“I had an appointment with my psychiatrist, and I... asked him to write me a letter, and emailed him or forwarded him an email from the surgery team that went over what the requirements for the letter were—the WPATH requirements. He was like okay; I’ll look at this. And then I got an email a couple of days later where he said that he didn’t know how to write it.” (Participant 4)* *“[S]he’s like I’ve actually never had a transgender patient before, which can’t be true, like statistically, if she’s been practicing for several years. Anyway, I’m like basically, all these people need your expert opinion on how trans I am, and this lady has never met a trans person before... And a few of the things she said were harmful and I had to address them once I got my therapist now.” (Participant 10)*
Benefits of mental healthcare received	*“It was just really good to have someone that really was first getting to know me as I was getting to know myself. So that was a really just affirming feeling, to have someone to talk to about some of my fears, some of my concerns and some of the challenges. And it was one of the first places where I started gradually coming out of my shell and presenting myself as myself. So, yeah, it was just … it was kind of like a little island. If my house was like a fortress of safety, it was a little safe island that I could go to away from that.” (Participant 6)* *“It has always been helpful for me. Sometimes it’s frustrating, but I think seeking help for mental health in general is extremely important for most people, most everyone, and especially for me. It is a necessary part of my life. I very literally and figuratively, but most importantly literally, could not live without mental health support.” (Participant 11)*

Two (14%) participants also expressed a feeling that they needed to “play the game” or adopt a particular narrative to obtain their documentation for surgery. As one participant described: “Something goes off in my head telling me not to say the truth, even though I am, to my therapist, because I know that there are specific answers to those questions that they will write the letter for... My gut feeling is that they want you to say that you’ve been transgender since birth, that you knew it intrinsically and hid it.” (Participant 10).

Patients had differing perspectives on the care that they received, with common themes being desire for more accessible, high quality mental health providers, desire for fewer administrative hurdles, and issues related to the expense associated with surgery and the pre-surgical process.

## Discussion

Overall, patients had differing and complex relationships with mental health care and the pre-surgical process. The incidence of comorbid psychiatric conditions was high in our participant group, exceeding the national average for these diagnoses but consistent with the known higher rates of depression, suicidality, and self-harm behaviours in transgender patients [5, 6, 13]. One participant summarized her perspective on this phenomenon during her interview: “The trans community doesn’t have mental health problems because they’re transgender. They have mental health problems because of society since the day they were born.” (Participant 10). Because of these findings, many take the position that significant mental health care and assessment is necessary before patients pursue life altering surgical interventions [14].

Overall the vast majority of participants, 93% did identify ways in which mental health care provided benefit to them and three of the five participants who reported seeking mental health care only to get referral letters for surgery continued to follow with a mental health care provider or planned to do so even after obtaining their documentation. Although there is evidence that a patient who does not willingly enter therapy has a weaker therapeutic relationship initially [15], these patients clearly found a real benefit in the mental health care they received.

Still, a large proportion of our participants reported significant concerns with the role of mental health care and the requirements set forth by WPATH and this has been previously reported in other literature [16–18]. The majority of our study participants (75%) described “preventing regret” in patients as a goal of the current WPATH SOC and the main intent of the two letter requirement but many participants expressed deep skepticism that a person would go on to regret gender affirmation following surgery. There were also participants in this study who found the process not only difficult to navigate but also “degrading.”

We interviewed study participants who felt that the process was “homophobic” or “traumatic” and impeded the pre-surgical process. Several participants reported that they felt the need to share a particular narrative to their mental health provider in order to obtain their letters for surgery. On the other hand, most study participants appreciated the need for supportive mental health care and found a personal benefit to the mental health care they received. This data indicates the need to adapt our current processes, and find the right balance between respecting patient autonomy and ensuring they receive safe, comprehensive care.

The strengths of this study lie in its qualitative, mixed methods approach and the high-quality data retrieved from participants. Several of the themes uncovered by this study were never directly asked about but arose spontaneously from participants during the interview process. This is also the first study to investigate these subject lines using this type of research methodology.

### Limitations

Limitations of our study include a small sample size because of quick saturation in themes achieved from the transcribed interviews conducted, as well as a homogenous study population. All participants were recruited from a single clinic and were overwhelmingly white and highly educated. Although this presents a significant bias in our study, this may reflect a larger trend where these surgeries and services are less accessible to patients who are non-white and less educated. This speaks to a much larger problem in the way these surgeries are made available to patients. There is also selection bias inherint in the study as not every patient who was eligible to participate elected to do so.

The WPATH SOC serves as an international guideline, but there is little data in the literature about how it is implemented and perceived in different countries. Our study population was from a single American center, which limits the generalizability of our data to an international cohort.

Additional limitations of this study include the lack of any transgender persons involved in study design or as part of the research team.

Lastly all participants in this study were transgender women who had already overcome the barriers to accessing care and were being scheduled for vaginoplasty. This design was intentional; however, the perspectives elicited in this study do not include those who never present to a gender clinic or those who are unable to qualify for gender-affirming surgery.

## Conclusion

There are many competing goals to balance when it comes to the guidelines for gender affirmation surgery: ensuring mental health care is received by those who need it, ensuring patients are appropriately prepared for surgery, and ensuring that the guidelines in place do not harm patients seeking treatment. This study looked directly to patients to share what their experiences and perspectives are when it comes to these competing interests and highlighted the importance of this type of research as we continue to adapt to patients’ needs.

While patients were overall understanding of the need to have mental health support during an often challenging time in their transition, our data points to a need to improve the quality of mental health care provided while reducing barriers to care and administrative requirements. This author group believes that a letter of support from a mental health care provider is not equivalent with supportive mental health care, and more needs to be done to ensure patients have access to necessary care without increasing administrative burden.

## Data Availability

The datasets generated and/or analyzed during the current study are not publicly available due to the sensitive nature of patient information included in the study, but deidentified data are available from the corresponding author on reasonable request.
